# Obituary

**Published:** 1854-08

**Authors:** 


### OBITUARY.

Died, in Perry, Illinois, July 11, 1854, of consumption, ISAAC NEWTON DAVIS, M.D , aged 26 years.

Dr. Davis was a graduate of Rush Medical College, and during his residence in this city had won for himself the confidence and esteem of all who knew him.

His good natural talents were cultivated by close observation and careful reading, while his heart was deeply imbued with the spirit of kindness and Christian Charity.

We knew him well, and as we watched his patience under suffering, and the fortitude and even cheerfulness with which he pursued the rugged paths of our science, conscious, meanwhile, that the slow destroyer was on his track, we learned to love him.

Modest and retiring in his manners, thorough in investigation, cool in judgment, and prompt in execution, with a warm heart and generous sympathies, he possessed those qualities eminently fitting him for the practice of the profession which he had chosen. While we sympathize with his afflicted friends, we recognise in his early death a loss to our profession and to science.

J.

---

Died, in Boston, July 1st, Dr. WALDO J. BURNETT, aged 26 years.

Dr. Burnett had won for himself an enviable reputation as a naturalist and physiologist. Although young in years, he had accomplished much for his profession and for natural history.

The Transactions of the American Association for the Advancement of Science have been enriched by several contributions from his pen. The principal work, however of his life was the essay published in the last volume of the Transactions of the American Medical Association, on "The Cell; its Physiology and Pathology."

It would seem hard that one so young and so gifted should die just as he was beginning to reap the reward of his labors, did we not believe that his active mind has found new and richer fields to explore, and that his heart, so full of love for nature, is already exulting in the apprehension of sublimer truths of the spirit-land.

